# Efficacy Study of Novel Diamidine Compounds in a *Trypanosoma evansi* Goat Model

**DOI:** 10.1371/journal.pone.0020836

**Published:** 2011-06-17

**Authors:** Kirsten Gillingwater, Carlos Gutierrez, Arlene Bridges, Huali Wu, Stijn Deborggraeve, Rosine Ali Ekangu, Arvind Kumar, Mohamed Ismail, David Boykin, Reto Brun

**Affiliations:** 1 Department of Medical Parasitology and Infection Biology, Swiss Tropical and Public Health Institute, Basel, Switzerland; 2 University of Basel, Basel, Switzerland; 3 Veterinary Faculty of the University of Las Palmas, Arucas, Gran Canaria, Spain; 4 School of Pharmacy, University of North Carolina, Chapel Hill, North Carolina, United States of America; 5 Department of Pathology, University of North Carolina, Chapel Hill, North Carolina, United States of America; 6 Department of Parasitology, Institute of Tropical Medicine, Antwerp, Belgium; 7 Institut National de Recherche Biomédicale, Kinshasa-Gombe, Democratic Republic of the Congo; 8 Department of Chemistry, Georgia State University, Atlanta, Georgia, United States of America; 9 Department of Chemistry, College of Science, King Faisal University, Hofuf, Saudi Arabia; Agency for Science, Technology and Research - Singapore Immunology Network, Singapore

## Abstract

Three diamidines (DB 75, DB 867 and DB 1192) were selected and their ability to cure *T. evansi* experimentally infected goats was investigated. A toxicity assessment and pharmacokinetic analysis of these compounds were additionally carried out. Goats demonstrated no signs of acute toxicity, when treated with four doses of 1 mg/kg/day (total dose 4 mg/kg). Complete curative efficacy of experimentally infected goats was seen in the positive control group treated with diminazene at 5 mg/kg and in the DB 75 and DB 867 groups treated at 2.5 mg/kg. Drug treatment was administered once every second day for a total of seven days. Complete cure was also seen in the group of goats treated with DB 75 at 1.25 mg/kg. DB 1192 was incapable of curing goats at either four-times 2.5 mg/kg or 1.25 mg/kg. Pharmacokinetic analysis clearly demonstrated that the treatment failures of DB 1192 were due to sub-therapeutic compound levels in goat plasma, whilst compound levels for DB 75 and DB 867 remained well within the therapeutic window. In conclusion, two diamidine compounds (DB 75 and DB 867) presented comparable efficacy at lower doses than the standard drug diminazene and could be considered as potential clinical candidates against *T. evansi* infection.

## Introduction

Aromatic diamidines bind to the minor groove of DNA and display a broad spectrum of antitrypanosomal activity [1], [2]. They exert their biological activity by primarily binding at A-T rich sites of DNA and then inhibiting one or more of the DNA dependent enzymes (such as topoisomerases or nucleases) or by directly impeding the transcription process. The selective binding to kinetoplastic DNA has also been suggested to play a vital role in the action of aromatic diamidines against pathogens [1]. Several novel diamidine analogues were found to be highly effective against *Trypanosoma evansi* [3], the causative agent of Surra, which presents itself as a fatal wasting disease in many domestic and wild animals, inhabiting South America, Africa and Asia [4]–[6]. Through mechanical transmission performed by biting flies (Tabanids), infection can quickly spread amongst close-living herds of cattle, water buffalo, horses and camels [7]. With the hindrance of resistant strains, unavailable and toxic drugs and the great economic losses involved, controlling this disease is a complex process [8], which could be simplified if alternative chemotherapeutic agents were obtainable. Since chemotherapy is the main form of control for Surra, existing drugs need to be replaced with improved compounds providing a more economical, effective and safer alternative against *T. evansi* [9].

*T. evansi* is considered endemic in certain areas of the Canary Islands, especially southern Gran Canaria. This was originally due to the importation of a male dromedary camel in 1997, from the neighbouring area of West Africa, where Surra is highly prevalent. Camels were primarily imported for labour purposes, but are nowadays principally raised for the tourism industry [10]. Seroprevalences in camels on the Canary Islands have been calculated to be around 4.8% to 9%, between 1997 and 1999 [11]. Although camels and horses are the major hosts affected on these islands, dissemination of the disease in other hosts has not yet been ruled out. Above all, goats appear to play an important role in disease epidemiology, especially as potential reservoir hosts [12. 13].

Three lead compounds (DB 75, DB 867 and DB 1192) were previously selected based on their *in vitro* activity and *in vivo* efficacy within a mouse model, their reduced potential for drug resistance, low cytotoxicity against mammalian cells and no toxicity in mice [3], [14]. Additionally, these compounds were selected due to their estimated cost-effective manufacture as possible clinical candidates against *T. evansi*.

Toxicity studies with these compounds have not yet been performed. Hence an experiment was conducted to determine whether the selected compounds produce signs of acute toxicity in goats, when administered at cumulative multiple doses of 1 mg/kg. Should these compounds exert no toxic effects at the tested compound dose, it may be possible to formulate a safe and successful treatment schedule to determine the efficacy of these diamidines against *T. evansi* infected goats.

The objectives of this study were the establishment of experimental infection of a local *T. evansi* strain in goats and the examination of chemotherapeutic effects of three selected lead compounds by comparing with diminazene aceturate (standard drug for *T. evansi* infection) in experimentally infected goats. Additionally, pharmacokinetic parameters were also examined for understanding the levels of drug that might be associated with efficacy and/or toxicity.

## Materials and Methods

### Ethics statement

The experimental protocol used was approved by the ethics committee for animal experimentation by the Veterinary Faculty of the University of Las Palmas on June 21, 2006 with the reference number 14/2006. This study was conducted under the strict guidelines set out by the FELASA for the correct implementation of animal care and experimentation.

### Trypanosome strain

The Canaries (Rubio) strain of *T. evansi* was used for goat infection in the efficacy and pharmacokinetic studies. This strain was originally isolated by K Gillingwater in 2006, from a 22 years old dromedary camel on Gran Canaria, Spain. The camel blood was immediately inoculated into mice and after a single mouse passage, used for experimental infection of the goats.

### Mice

Female NMRI mice weighing between 25 – 30 g were used for strain isolation. All mice were specific pathogen free (SPF) and were maintained in standard Macrolon type II cages, at 22°C and with a relative humidity of 60 – 70%. Water and pelleted food was provided *ad libitum*. All *in vivo* experiments performed complied with the regulations set out by the Swiss Federal Veterinary Office.

### Goats

The toxicity, efficacy and pharmacokinetic studies all took place within the Veterinary Hospital of the University of Las Palmas in Arucas, Gran Canaria, between June 2006 and January 2007. In total, 33 female Canarian goats, weighing between 20 – 52 kg and no less than six months old were purchased from a local dairy farmer. The goats were placed inside open pens and allowed to acclimatise for a week. Thereafter, the goats were given numbered ear tags, weighed and checked for *T. evansi* infection using the diagnostic methods described within this study. Any goats showing positive results for previous *T. evansi* infection were removed from the project (in this case, two goats were removed). The remaining goats were then randomly divided into test groups, given oxytetracycline against nasal exudate and ivermectin as a standard anti-helminthic, before being placed into fly-proof pens. Throughout the study period, the goats were given food (pelleted nutrients, dried corn and hay), three times a day and water was provided *ad libitum*.

### Test compounds and stock solutions

Diminazene aceturate (D-7770, Sigma, St Louis, MO, USA), was used as the standard positive control drug. The selected lead diamidine compounds were previously synthesised in the laboratory of Professor David Boykin. The compounds DB 75, DB 867 and DB 1192 were used as the test compounds in these goat studies based on their previous *in vitro* and *in vivo* activity and safety profile [3], [14].

The 10 mg/ml stock solutions of DB 75, DB 867 and DB 1192 were prepared in 10% DMSO on the day of the first administration and were stored at 4°C. For the control compound, a 50 mg/ml stock solution of diminazene aceturate was also prepared in 10% DMSO.

### Toxicity trials

For the toxicity trials, six goats were divided into three groups of two. Each group was allocated to one selected diamidine compound. Both goats in each group undertook four compound applications of 1 mg/kg, given at two hour intervals, over a total six hour period as intramuscular injection into the lower third region of the neck muscles. The goats were individually observed and assessed for signs of acute toxicity.

### Diagnostic tests

Various diagnostic tests were used to evaluate the curative efficacy of the compounds, over a five month follow up period. The haematocrit centrifugation technique [15], was utilised as the standard parasitological test. The serological CATT/*T. evansi* test was performed to determine the presence or absence of antibodies [16]. As a validating molecular based technique, a mini-exon 18S ribosomal PCR method designed for the detection of parasite DNA was also applied [17].

### Experimental infection and treatment

Fresh camel blood (400 µl) was collected from the jugular vein of a camel and 200 µl volumes inoculated (i.p.) into two mice. Once a parasitaemia had established, blood was collected via cardiac puncture and mixed with phosphate buffered saline with glucose (PSG) to provide the *T. evansi* suspension used for experimental goat infection. Thereafter, all 25 goats were experimentally infected intravenously (i.v.), with 10^6^ parasites/goat.

Goats were divided into 8 groups, where group 1 contained four goats whilst group's 2 to 8 each contained three goats. After confirmation of infection, treatment was applied to the various groups of goats as follows: untreated control, given no compound treatment (Group 1); positive control, given 5 mg/kg diminazene aceturate (Group 2); given 2.5 mg/kg and 1.25 mg/kg of DB 75 (Group 3 and 6), of DB 867 (Group 4 and 7) and of DB 1192 (Group 5 and 8), respectively. All test compounds were administered intra-muscularly with four applications, on days 32, 34, 36 and 38 post-infection. Thereafter, follow up diagnostic methods were carried out for five months post treatment.

### Measurement of drug levels and pharmacokinetic parameters

Blood samples (4 ml volumes) were taken for pharmacokinetic analysis at the following time points of 0.50, 1, 2, 4, 8, 24, 48, 96 and 192 hours after the administration of the fourth compound injection, using sterile Vacutainer® (Becton Dickinson, Plymouth, UK) tubes, containing the anticoagulant, ethylene diamine tetra acetic acid (EDTA). In addition, blood samples were taken shortly before each compound injection was applied, to determine the trough levels. All samples were centrifuged at 3000 *g* for ten minutes. Subsequently, the plasma was removed, placed into sterile polypropylene cryotubes and stored away from light at −80°C.

Mass spectrometry analysis was performed on all plasma samples using 25 µl aliquots. Calibrators were prepared separately for each analyte. Stock solutions of diminazene, DB 75, DB 867 and DB1192 were prepared in water. Curves were then prepared and analysed in duplicate, at both the beginning and the end of the analytical run. Concentrations of diminazene, DB 75, DB 867 and DB 1192 were determined by Liquid Chromatography/Triple Quadrupole Mass Spectrometry (HPLC/MS-MS). The pharmacokinetic parameters were determined using Microsoft® Excel software, with a functional set of add-ins applied, together with a graphical programme (GraphPad Prism 5, GraphPad Software Inc, USA).

## Results

### Establishment of experimental infection in goats

Successful establishment of a *T. evansi* goat model of infection was achieved through infection with a local *T. evansi* strain, originally isolated from a camel. The parasitaemia observed in the infected camel was sporadic and not always detectable. Therefore, several mice were inoculated with blood from the camel to enable propagation of a sufficient number of trypanosomes, allowing each goat to be initially inoculated with 10^6^ parasites. Inoculation number was determined using a haemocytometer.

### Toxicity of test compounds

The signs of acute toxicity seen in the goats for DB 75, DB 867 and DB 1192 are shown in Table 1. Signs of acute toxicity assessed, included tremors, lacrimation, excess salivation, irritation (scratching at injection site or general discomfort), excess urination, diarrhoea, hypertension and hypotension. For goats treated with DB 75 and DB 867, some irritation was observed by means of scratching at the injection site. These goats additionally showed a short phase of discomfort, immediately after each compound was applied. These symptoms disappeared after ten minutes and the goats returned to their normal behaviour.

**Table 1:** Signs of acute toxicity investigated after the i.m. application of DB 75, DB 867 and DB 1192 in a multiple compound application (4×1 mg/kg) toxicity trial.

Group	Lead compound	Toxicitytrial	Goat number	Signs of acute toxicity*							
				Hypo	Hyper	(L)	(S)	(Tr)	(Ir)	(U)	(D)
1	DB 75	multiple	0823	x	x	x	x	x	✓	x	x
			0865	x	x	x	x	x	✓	x	x
2	DB 867	multiple	0866	x	x	x	x	x	✓	x	x
			0850	x	x	x	x	x	✓	x	x
3	DB 1192	multiple	0818	x	x	x	x	x	x	x	x
			0810	x	x	x	x	x	x	x	x

The presence or absence of each sign is depicted as a (✓) or as a (x), respectively.

*Signs of acute toxicity: Hypo: *Hypotension*; Hyper: *Hypertension*; (L): *Lacrimation*; (S): *Salivation* (excess); (Tr): *Tremors*; (Ir): *Irritation*; (U): *Urination* (excess); (D): *Diarrhoea*.

### Efficacy of test compounds against infection

The results obtained from the efficacy study by three diagnostic tests, the haematocrit centrifugation technique (HCT), the card agglutination test (CATT/*T. evansi*) and the polymerase chain reaction (PCR), are shown in Table 2, 3 and 4, respectively. Infection was confirmed positive in all goats before treatment commenced. The control group (group 1), which received no drug treatment, remained positive for the HCT, strongly positive (+++) for the CATT/*T. evansi* and positive for the PCR method during the complete five month follow up period. Two of the four control goats died on days 114 and 156 post infection. The positive control group (group 2), treated with 5 mg/kg diminazene aceturate, demonstrated negative results for both the HCT and the PCR methods for the whole duration of the study. The CATT/*T. evansi* serological test demonstrated a gradual decrease from strongly positive (+++) to negative (−) for 2 out of the 3 goats.

**Table 2:** A five month follow up of an efficacy study for a *T. evansi* experimentally infected goat model, using the haematocrit centrifugation technique (HCT), after treatment with Diminazene, DB 75, DB 867 or DB 1192 at various compound doses.

Group	Compound Treatment	Goat No.	Follow up goat efficacy using haematocrit centrifugation technique (HCT) post treatment (in days)																
			−7	1	7	14	21	28	35	42	49	56	70	84	98	112	126	140	150
1	Control	2255	+	+	+	+	+	+	+	+	+	+	+	+	+	+	+	+	+
		7397	+	+	+	+	+	+	+	+	+	+	+	+	+	+	*D*		
		7374	+	+	+	+	+	+	+	+	+	+	+	+	+	+	+	+	+
		7314	+	+	+	+	+	+	+	+	+	+	+	*D*					
2	Diminazene	1773	+	−	−	−	−	−	−	−	−	−	−	−	−	−	−	−	−
	*5* *mg/kg*	7396	+	−	−	−	−	−	−	−	−	−	−	−	−	−	−	−	−
		7388	+	−	−	−	−	−	−	−	−	−	−	−	−	−	−	−	−
3	DB 75	7390	+	−	−	−	−	−	−	−	−	−	−	−	−	−	−	−	−
	*2.5* *mg/kg*	7324	+	−	−	−	−	−	−	−	−	−	−	−	−	−	−	−	−
		5406	+	−	−	−	−	−	−	−	−	−	−	−	−	−	−	−	−
4	DB 867	7319	+	−	−	−	−	−	−	−	−	−	−	−	−	−	−	−	−
	*2.5* *mg/kg*	5674	+	−	−	−	−	−	−	−	−	−	−	−	−	−	−	−	−
		7386	+	−	−	−	−	−	−	−	−	−	−	−	−	−	−	−	−
5	DB 1192	7379	+	−	−	+	*Rem*												
	*2.5* *mg/kg*	7387	+	−	−	+	*Rem*												
		7361	+	−	−	−	−	−	*D*										
6	DB 75	5622	+	−	−	−	−	−	−	−	−	−	−	−	−	−	−	−	−
	*1.25* *mg/kg*	7366	+	−	−	−	−	−	−	−	−	−	−	−	−	−	−	−	−
		7369	+	−	−	−	−	−	−	−	−	−	−	−	−	−	−	−	−
7	DB 867	7372	+	−	−	−	−	−	−	−	−	−	−	−	−	−	−	−	+
	*1.25* *mg/kg*	7341	+	−	−	−	−	−	−	−	−	−	−	−	−	−	−	−	+
		7339	+	−	−	−	−	−	−	−	−	−	−	−	−	−	−	−	+
8	DB 1192	7384	+	−	−	+	*Rem*												
	*1.25* *mg/kg*	7383	+	−	+	+	*Rem*												
		7360	+	−	−	+	*Rem*												

The results are depicted as negative (−) or positive (+) for motile trypanosomes observed in goat blood samples, along with goats, which died (*D*) or were removed from the project (*Rem*) during the study period.

**Table 3:** A five month follow up of an efficacy study for a *T. evansi* experimentally infected goat model, using a card agglutination test (CATT/*T. evansi*), after treatment with Diminazene, DB 75, DB 867 or DB 1192 at various compound doses.

Group	Compound Treatment	Goat No.	Follow up goat efficacy using a card agglutination test (CATT/*T. evansi*) post treatment (in days)																
			−7	1	7	14	21	28	35	42	49	56	70	84	98	112	126	140	150
1	Control	2255	+++	+++	+++	+++	+++	+++	+++	+++	+++	+++	+++	+++	+++	+++	+++	+++	+++
		7397	+++	+++	+++	+++	+++	+++	+++	+++	+++	+++	+++	+++	+++	+++	*D*		
		7374	+++	+++	+++	+++	+++	+++	+++	+++	+++	+++	+++	+++	+++	+++	+++	+++	+++
		7314	+++	+++	+++	+++	+++	+++	+++	+++	+++	+++	+++	*D*					
2	Diminazene	1773	+++	+++	+++	++	++	++	+	+	+	−	−	−	−	−	−	−	−
	*5* *mg/kg*	7396	+++	+++	+++	+++	+++	+++	+++	++	++	++	++	++	++	++	++	++	++
		7388	+++	++	−	−	−	−	−	−	−	−	−	−	−	−	−	−	−
3	DB 75	7390	+++	+++	+++	+++	+++	+++	++	++	++	+	+	+	+	+	+	+	+
	*2.5* *mg/kg*	7324	+++	+++	+++	+++	+++	+++	++	+	+	−	−	−	−	−	−	−	−
		5406	+++	+++	+++	+++	+++	+++	++	++	++	++	++	+	+	+	+	+	+
4	DB 867	7319	+++	++	++	−	−	−	−	−	−	−	−	−	−	−	−	−	−
	*2.5* *mg/kg*	5674	+++	+++	+++	++	+	+	+	+	+	+	+	−	−	−	−	−	−
		7386	+++	+++	+++	+++	++	++	+	+	+	+	+	+	+	+	+	+	+
5	DB 1192	7379	+++	+++	+++	+++	*Rem*												
	*2.5* *mg/kg*	7387	+++	+++	+++	+++	*Rem*												
		7361	+++	+++	++	++	+++	+++	*D*										
6	DB 75	5622	+++	+++	++	+	+	+	+	+	+	−	−	−	−	−	−	−	−
	*1.25* *mg/kg*	7366	+++	+++	+++	+++	+++	+++	++	++	++	++	++	++	++	++	++	++	++
		7369	+++	+++	+++	+++	+++	+++	+++	++	+	+	−	−	−	−	−	−	−
7	DB 867	7372	+++	+++	+++	+++	+++	+++	+++	+++	+++	+++	+++	++	++	++	++	++	+++
	*1.25* *mg/kg*	7341	+++	+++	++	++	++	++	++	++	++	+	+	+	+	+	+	++	+++
		7339	+++	+++	++	+	+	+	+	+	+	+	+	+	+	+	+	++	+++
8	DB 1192	7384	+++	+++	+++	+++	*Rem*												
	*1.25* *mg/kg*	7383	+++	+++	+++	+++	*Rem*												
		7360	+++	+++	+++	+++	*Rem*												

Results are depicted as strongly positive (+++), intermediate (++), weak (+) or negative (−) for agglutination, when goat plasma was mixed with an antigenic reagent, along with goats, which died (*D*) or were removed from the project (*Rem*) during the study period.

**Table 4:** A five month follow up of an efficacy study for a *T. evansi* experimentally infected goat model, using the polymerase chain reaction (PCR), after treatment with Diminazene, DB 75, DB 867 or DB 1192 at various compound doses.

Group	Compound Treatment	Goat No.	Follow up goat efficacy using polymerase chain reaction (PCR) post treatment (in days)																
			−7	1	7	14	21	28	35	42	49	56	70	84	98	112	126	140	150
1	Control	2255	+	+	+	+	+	+	+	+	+	+	+	+	+	+	+	+	+
		7397	+	+	+	+	+	+	+	+	+	+	+	+	+	+	*D*		
		7374	+	+	+	+	+	+	+	+	+	+	+	+	+	+	+	+	+
		7314	+	+	+	+	+	+	+	+	+	+	+	*D*					
2	Diminazene	1773	+	−	−	−	−	−	−	−	−	−	−	−	−	−	−	−	−
	*5* *mg/kg*	7396	+	−	−	−	−	−	−	−	−	−	−	−	−	−	−	−	−
		7388	+	−	−	−	−	−	−	−	−	−	−	−	−	−	−	−	−
3	DB 75	7390	+	−	−	−	−	−	−	−	−	−	−	−	−	−	−	−	−
	*2.5* *mg/kg*	7324	+	−	−	−	−	−	−	−	−	−	−	−	−	−	−	−	−
		5406	+	−	−	−	−	−	−	−	−	−	−	−	−	−	−	−	−
4	DB 867	7319	+	−	−	−	−	−	−	−	−	−	−	−	−	−	−	−	−
	*2.5* *mg/kg*	5674	+	−	−	−	−	−	−	−	−	−	−	−	−	−	−	−	−
		7386	+	−	−	−	−	−	−	−	−	−	−	−	−	−	−	−	−
5	DB 1192	7379	+	−	+	+	*Rem*												
	*2.5* *mg/kg*	7387	+	−	−	+	*Rem*												
		7361	+	−	−	−	−	+	*D*										
6	DB 75	5622	+	−	−	−	−	−	−	−	−	−	−	−	−	−	−	−	−
	*1.25* *mg/kg*	7366	+	−	−	−	−	−	−	−	−	−	−	−	−	−	−	−	−
		7369	+	−	−	−	−	−	−	−	−	−	−	−	−	−	−	−	−
7	DB 867	7372	+	−	−	−	−	−	−	−	−	−	−	−	−	−	+	+	+
	*1.25* *mg/kg*	7341	+	−	−	−	−	−	−	−	−	−	−	−	−	−	+	+	+
		7339	+	−	−	−	−	−	−	−	−	−	−	−	−	−	−	+	+
8	DB 1192	7384	+	+	+	+	*Rem*												
	*1.25* *mg/kg*	7383	+	+	+	+	*Rem*												
		7360	+	+	+	+	*Rem*												

The results are depicted as negative (−) or positive (+) for the presence of parasite DNA in goat blood samples, along with goats, which died (*D*) or were removed from the project (*Rem*) during the study period.

Goats treated with DB 75 at either 2.5 mg/kg or 1.25 mg/kg (groups 3 and 6, respectively) demonstrated negative results for both the HCT and PCR methods for the complete five month follow up. For the 2.5 mg/kg group (group 3), goats showed a strong positive reaction (+++) towards the CATT/*T. evansi* test until day 35 post treatment, when a gradual decrease occurred leaving one goat negative and two goats weakly positive (+). A gradual decrease in antibody reaction was also seen for the 1.25 mg/kg group (group 6) with two goats completely negative and one showing an intermediate result (++) at the end of the follow up period. A similar pattern was seen in goats treated with DB 867 at 2.5 mg/kg (group 4), where all three animals remained negative for the HCT and the PCR methods during all five months. The same situation was recorded for the goats treated with DB 867 at 1.25 mg/kg (group 7) for the HCT and PCR methods, until day 126 post treatment, when the PCR became positive for two out of three goats. By day 140 post treatment, all three goats had relapsed and tested positive with PCR. On day 150 post treatment, trypanosomes were visibly observed in all three goats. The serological results for DB 867 treated goats showed a good correlation with the other two diagnostic tests used. At 2.5 mg/kg treatment dose (group 4), the CATT/*T. evansi* results gradually decreased throughout the trial, with two negative goats and one weakly positive (+) goat at the end. In contrast, the 1.25 mg/kg treated group (group 7) demonstrated a gradual decrease in CATT/*T. evansi* results by day 84 post treatment. Thereafter, an increase in reaction intensity was seen from day 140 post treatment onwards, as all three goats relapsed.

For DB 1192 treated goats given the 2.5 mg/kg dose (group 5), the HCT remained negative for just under 2 weeks before trypanosomes reappeared in two of the three goats. These two goats were then removed from the study. The third goat remained HCT negative, but later died on day 35 post treatment. The PCR method detected positive reactions in all three goats on days 7, 14 and 21 post treatment, respectively. The CATT/*T. evansi* reaction remained strongly positive (+++) for all three goats. For the 1.25 mg/kg treated goats (group 8), one goat became HCT positive after just one week post treatment. By day 14 post treatment, all three goats demonstrated trypanosomes in the blood and hence were removed from the study.

### Concentration of test compounds in blood

**Figure 1:**
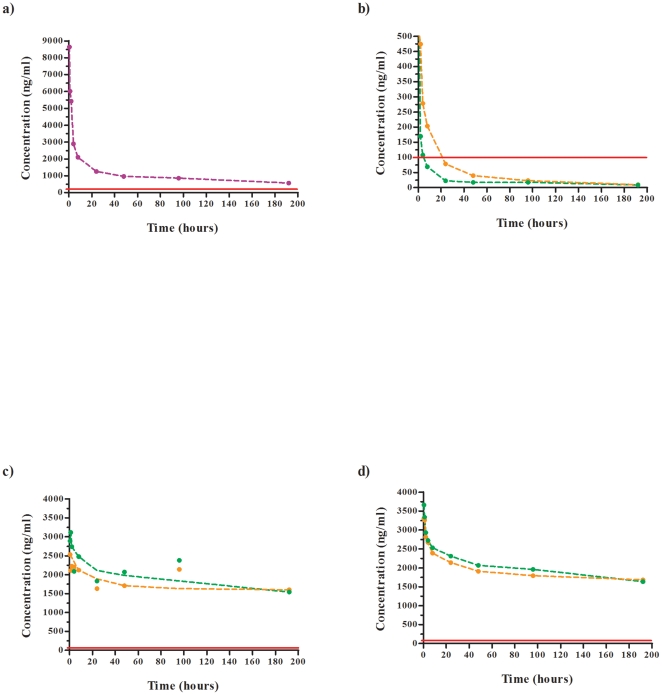
Plasma profiles obtained for the standard drug and test compounds used in a *T. evansi* experimental goat model of infection. Drug plasma concentrations of a) diminazene at 5 mg/kg (purple), b) DB 1192 at 2.5 mg/kg (orange) and 1.25 mg/kg (green), c) DB 75 at 2.5 mg/kg (orange) and 1.25 mg/kg (green) and d) DB 867 at 2.5 mg/kg (orange) and 1.25 mg/kg (green) are depicted. Horizontal red line shows ten-times the IC_50_ for that respective compound.

The compound plasma levels can be seen in Figure 1 for a) the standard drug, diminazene, b) DB 1192, c) DB 75 and d) DB 867. The plasma samples taken were analysed by HPLC/MS-MS to determine the drug concentrations (in ng/ml). From these values, the averages from the experimental goats in each group were calculated and drug concentration versus time curves were plotted with a line of best fit applied, corresponding to either the high or low compound dose tested.

For the standard control drug (diminazene), the *in vitro* IC_50_ value obtained for 72 hours is 12.5 ng/ml, with an estimated MIC of 50 ng/ml [14]. At 72 hours after the end of treatment, the concentration of diminazene in goat plasma was 1112 ng/ml, which is a 22-fold greater concentration than the 50 ng/ml MIC value. Even after 192 hours, the diminazene concentration in goat plasma was 568 ng/ml, which still remains well within the therapeutic range.

Through previous *in vitro* testing of DB 1192 against *T. evansi*, an IC_50_ value of 10.5 ng/ml was obtained for a 72 hour assay. The minimal inhibitory concentration (MIC) of such a compound was estimated at least four times greater than the IC_50_, so for DB 1192, the estimated MIC would be around 40 ng/ml [14]. To establish curative efficacy of an infected animal, the level of the compound in plasma must exceed the MIC for a certain time period. If the compound level in plasma falls below the MIC threshold too quickly, the compound provides only sub-therapeutic concentrations, which in turn will be insufficient to remove the parasites. At 72 hours, the compound concentration in the plasma was only 32 ng/ml. This value is already below the MIC threshold to ensure successful therapeutic activity of DB 1192. At 192 hours after the final compound injection was applied to goats treated with 2.5 mg/kg, the compound concentration in plasma was only 10 ng/ml. Thus, there is clear evidence why DB 1192 could not cure at 2.5 mg/kg goats experimentally infected with *T. evansi*. Furthermore, this relates to the lower dose of DB 1192 tested (1.25 mg/kg), where compound concentrations in goat plasma at 72 hours were only 18 ng/ml as compared to 40 ng/ml value for the MIC.

A similar pattern was seen for DB 75 at 2.5 mg/kg and 1.25 mg/kg as that of the standard drug, where the plasma concentrations measured at 72 hours were 1923 ng/ml and 2223 ng/ml, respectively. DB 75 has an IC_50_ of 2.3 ng/ml at 72 hours and an estimated MIC of 9.2 ng/ml [14]. At 192 hours, the plasma concentrations of DB 75 for both doses were above 1500 ng/ml and well within the therapeutic range and thus explaining effective cure. Treatment with even lower doses than 1.25 mg/kg are still likely to be curative.

In the case of DB 867, the *in vitro* IC_50_ at 72 hours was previously determined as 1.7 ng/ml and the MIC as 6.8 ng/ml [14]. Both the 2.5 mg/kg and 1.25 mg/kg DB 867 treated goat's demonstrated compound concentrations in plasma at 72 hours above 1850 ng/ml. After 192 hours, the compound concentration levels were still within the therapeutic range. Goats treated with the higher dose (2.5 mg/kg) of DB 867 were cured, whereas goats treated with the lower dose of 1.25 mg/kg were seen to relapse after 4.5 months post treatment.

### Pharmacokinetic parameters

The maximum drug concentration (C_max_) obtained for the control drug diminazene was ≥8637 ng/ml. In comparison, DB 75 and DB 867 produced C_max_ values of ≥2530/≥3115 ng/ml and ≥3310/≥3663 ng/ml for the 2.5/1.25 mg/kg treatment doses tested, respectively (Table 5). At these C_max_ values, compound levels remained well over the therapeutic threshold and no significant correlation was seen between the two doses applied. DB 1192 demonstrated low C_max_ values at both doses and fell below the therapeutic threshold within the first 24 hours post treatment. In addition, the DB 1192 C_max_ value of ≥1563 ng/ml at 2.5 mg/kg was approximately double that of the C_max_ value of ≥836 ng/ml at the lower dose of 1.25 mg/kg. The half lives (T ½) of DB 75 and DB 867 were found to be incredibly long as compared to that of diminazene. As expected, DB 1192 had a short half life at the ineffective doses tested in this study.

**Table 5:** Pharmacokinetic parameters determined for a treatment schedule of four applications of diminazene (at 5 mg/kg), DB 75, DB 867 and DB 1192 (at 2.5 mg/kg and 1.25 mg/kg), based on average plasma concentration levels taken from *T. evansi* experimentally infected goats.

Parameter	Diminazene	DB 75		DB 867		DB 1192	
	*5* *mg/kg*	*2.5* *mg/kg*	*1.25*	*2.5* *mg/kg*	*1.25*	*2.5*	*1.25*
C_max_ (ng/ml)	≥8637	≥2530	≥3115	≥3310	≥3663	≥1563	≥836
AUC	193589	358215	394305	360895	381604	9861	4809
MRT (hrs)	70.7	93.4	89.5	90.0	87.9	38.4	52.3
T_1/2_ (hrs)	59.1	473.7	279.3	221.4	202.5	30.5	37.0

## Discussion

In the present study, a constant detectable parasitaemia could be achieved (which is prerequisite for efficacy trials) by using a less virulent parasite strain (endemic in the Canary Islands) at a higher inoculation dose (10^6^ parasites/goat). Once the goat model of infection was established, the efficacy study could be conducted to investigate the curative potential of the three lead diamidines.

Complete curative efficacy was seen for the whole five month follow up period in the positive control group, treated with 5 mg/kg of diminazene. Additional complete curative efficacy was demonstrated in the goats treated with both 2.5 mg/kg and 1.25 mg/kg doses of DB 75. It may even be possible that this compound could cure *T. evansi* infected goats at a lower dose than 1.25 mg/kg. With DB 867, curative efficacy was seen in the higher treatment dose (2.5 mg/kg) while for the lower (1.25 mg/kg) dose a relapse was seen during the final month of the follow up period. Since the goats were housed in fly-proof facilities, the potential risk of re-infection is small, together with the knowledge that the control goats (which underwent no treatment) were also housed in a separate fly-proof pen within the research building. On the other hand, DB 1192 was not effective at either dose tested and the goats became parasitaemic just one month post treatment.

The pharmacokinetic study was performed to collect information for DB 75, DB 867 and DB 1192 within infected goats and to determine the pharmacokinetic profiles for each compound, including the standard drug, diminazene. By falling below the therapeutic threshold level (as did DB 1192), we can clearly visualise treatment failures due to inadequate compound concentrations in the blood. DB 75 and DB 867 appear to accumulate within the body, as determined through their long half lives. Since no differences were seen between the high (2.5 mg/kg) and low (1.25 mg/kg) doses tested, it may be that a point of saturation within the body was reached. If a higher dose is administered, a larger amount of compound will be bound to tissue, yet the amount free in the body may be the same. However, it is difficult to support such a hypothesis based solely on plasma concentration data and one would therefore require more in-depth research into the accumulation and tissue-binding properties of diamidines in general as well as the investigation of any injection-site residues.

The phenomenon of relapse cases in both human and animal trypanosomiasis is not fully understood and many factors, such as drug resistant strains, reinfection or insufficient drug levels at a given site of the body, can be attributed as possible explanations for this [18]. Although there are rare reported cases where *T. evansi* has established CNS infections [19], the extent to which these infections contribute to overall disease morbidity and mortality is unknown. Based on their dicationic nature, diamidines are not capable of crossing the blood-brain barrier and hence would not be effective in a CNS infection. If trypanosomes have an ability to enter compartments (for example, cartilage), which have a reduced blood flow, then insufficient drug levels may permeate these compartments, hence “hidden” trypanosomes will not be exposed sufficiently to the drug, enabling them to survive. Thereafter, once the drug within the body has been eliminated completely, these trypanosomes would reinvade the bloodstream and produce the high parasitaemia observed in the goats after 4.5 months post treatment with DB 867 at 1.25 mg/kg.

In conclusion, this efficacy trial into *T. evansi* infected goats has enabled three selected lead diamidine compounds to be evaluated, of which two (DB 75 and DB 867) demonstrated comparable efficacy to the standard drug (diminazene aceturate). DB 75 was equally effective at a quarter of the dose used (1.25 mg/kg) and possibly even at lower doses, than diminazene and DB 867 was equally effective at half the dose used (2.5 mg/kg). DB 75 and DB 867 warrant further investigation as potential clinical candidates against *T. evansi* infection.
